# Simultaneous Endoscopic Endonasal Decompression of the Optic Canal, Superior Orbital Fissure, and Proper Orbital Apex for Traumatic Orbital Apex Syndrome: Surgical Anatomy and Technical Note

**DOI:** 10.3389/fsurg.2021.811706

**Published:** 2022-01-21

**Authors:** Jianfeng Liu, Jianhui Zhao, Yibei Wang, Zhijun Wang, Rui Li, Zhongyan Chen, Yu Zhao, Jun Han, Dazhang Yang

**Affiliations:** ^1^Department of Otolaryngology-Head and Neck Surgery, China-Japan Friendship Hospital, Beijing, China; ^2^Department of Ophthalmology, China-Japan Friendship Hospital, Beijing, China; ^3^Department of Neurosurgery, China-Japan Friendship Hospital, Beijing, China

**Keywords:** superior orbital fissure, orbital apex syndrome, traumatic, endonasal approach, optic nerve, decompression, convergence prominence

## Abstract

**Objectives:**

Traumatic orbital apex syndrome (TOAS) is an uncommon but severe ocular complication of craniomaxillofacial fracture. The optimal surgical strategy for TOAS has not been determined. To investigate the endoscopic anatomy of the orbital apex region, propose a protocol for simultaneous endoscopic endonasal decompression of the optic canal, superior orbital fissure, and proper orbital apex (EEDCFA) for TOAS and report its use in two patients.

**Methods:**

An endoscopic endonasal approach was utilized to dissect the orbital apex region in two silicon-injected adult cadaveric heads. The details of the procedure used for decompression of the orbital apex were determined. The effects of this procedure were determined in two patients with TOAS who underwent simultaneous decompression of the optic canal, superior orbital fissure, and proper orbital apex.

**Results:**

The orbital apex consisted of three portions, the contents of the optic canal superomedially; the contents of the superior orbital fissure inferolaterally; and the converging portion, or proper orbital apex, anteriorly. From an endoscopic endonasal approach, the optic nerve, superior orbital fissure, and orbital apex convergence prominences were found to form a π-shaped configuration. This π-shaped configuration was indicative of the orbital apex and was an important landmark for decompression of the orbital apex. Endonasal decompression of the orbital apex in the two patients resulted in the satisfactory recovery of extraocular mobility, with no surgical complications.

**Conclusions:**

EEDCFA is feasible, effective, and safe for patients with TOAS caused by direct compression of displaced fracture segments. The π-shaped configuration is a valuable landmark for EEDCFA.

## Introduction

Traumatic orbital apex syndrome (TOAS) is an uncommon but severe ocular complication of craniomaxillofacial fracture, characterized by injuries to cranial nerves II, III, IV, V, and VI. From an anatomic perspective, trauma usually involves both the superior orbital fissure and the optic canal. Although, surgical decompression is a treatment of choice. The optimal surgical strategy for TOAS has not yet been determined. Methods described to date for patients with TOAS include endoscopic endonasal decompression of both the optic canal and proper orbital apex (1) and an open microsurgical approach involving decompression of both the superior orbital fissure and the optic canal (2). It is unclear, however, whether better clinical outcomes would result from simultaneous complete decompression of the superior orbital fissure, optic nerve, and the proper orbital apex. Surgical decompression of the superior orbital apex may be an option for some patients (2–5), but the use of an endoscopic endonasal approach for decompression of the superior orbital fissure or orbital apex is rare (6). To the best of our knowledge, no study to date has described the use of simultaneous endoscopic endonasal decompression of the superior orbital fissure, optic canal, and proper orbital apex in patients with TOAS in the English literature. In addition, although the microscopic anatomy of the superior orbital fissure and orbital apex has been described (7–9), few reports have assessed the orbital apex endoscopically (10–12).

This study was, therefore, designed to investigate the endoscopic anatomy of the orbital apex region, focusing on the interrelationships of the superior orbital fissure, optic nerve, and proper orbital apex. Based on these anatomic findings, a protocol was designed for the simultaneous endoscopic endonasal decompression of the optic canal, superior orbital fissure, and proper orbital apex (EEDCFA) and the benefits of this protocol were tested in two patients with TOAS.

## Materials and Methods

The orbital apex in two silicon-injected adult cadaveric heads (four sides) was approached *via* the endoscopic endonasal transethmoidal transsphenoidal route combined with the trans-septo-transsphenoidal route. The orbital apex was dissected and its detailed anatomy was determined using the nasal endoscope.

In addition, two patients with TOAS caused by direct compression from the displaced fracture segments were evaluated. The orbital apex in each was evaluated pre-operatively by CT scanning. The two patients underwent EEDCFA. Recovery was evaluated post-operatively by determining extraocular motility and visual acuity.

Institution Review Board ethics approval was obtained and patients consented to publication of their images.

## Results

### Osseous Anatomy of the Orbital Apex

**Figure 1:**
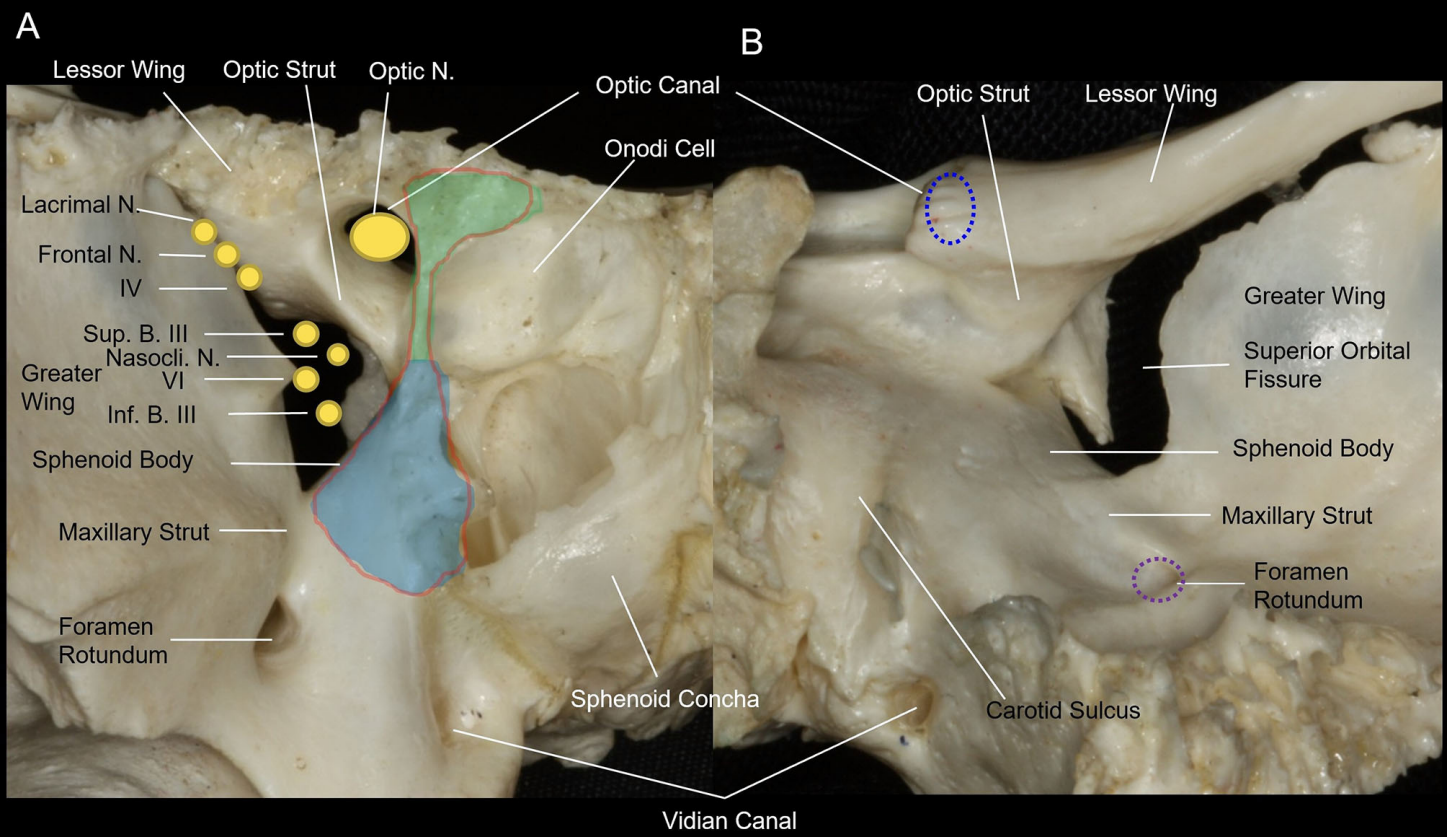
Anterior and posterior views of the osseous anatomy of the orbital apex of the sphenoid bone. **(A)** Anterior view of the osseous orbital apex of the right side. The osseous optic apex is comprised of the optic canal superomedially and the superior orbital fissure inferolaterally. The medial wall of the orbital apex is a part of the lateral wall of the sphenoid sinus. The contents of the superior orbital fissure are separated from the optic nerve and ophthalmic artery, which pass through the optic canal, by the optic strut superiorly. In addition, they are separated from the maxillary division of the trigeminal nerve that courses by the foramen rotundum by the maxillary strut inferiorly. The endoscopic endonasal approach for optic nerve decompression requires removal of the medial wall of the optic canal from the lateral roof of the sphenoid sinus superiorly to the lateral opticocarotid recess inferiorly, and from the lateral opticocarotid recess posteriorly to the optic tubercle anteriorly (light green area). The endoscopic endonasal approach for decompression of the superior orbital fissure requires removal of the medial wall of the superior orbital fissure, from the lateral opticocarotid recess superiorly to the maxillary strut inferiorly and from the cavernous sinus posteriorly to the junction of the sphenoid sinus and the posterior ethmoid sinus anteriorly (light blue area). The endoscopic endonasal approach for decompression of the orbital apex requires removal of the medial wall of the optic canal and the medial wall of the superior orbital fissure (red outline area). **(B)** Posterior view of the osseous orbital apex of the right side. The optic nerve and ophthalmic artery, which pass through the optic canal, are separated from the contents of the superior orbital fissure by the optic strut superiorly; and the maxillary division of the trigeminal nerve, which courses by the foramen rotundum, is separated from the contents of the superior orbital fissure by the maxillary strut inferiorly. The lower margin of the fissure is formed by the junction of the greater wing with the body of the sphenoid and is located at the level of the lower edge of the cavernous sinus and the floor of the middle fossa.

The apex of the orbit is located in the orbit posteriorly, thus forming a transition zone between the orbit and intracranial structures. The orbital apex consists of three portions: the contents of the optic canal superomedially; the contents of the superior orbital fissure inferolaterally; and the convergence portion, or proper orbital apex, anteriorly, in which the contents of the optic canal and the superior orbital fissure converge into the orbit. The superior orbital fissure and the optic canal form the posterior osseous orbital apex (Figures 1A,B). The superior orbital fissure is situated between the greater and lesser wings and the body of the sphenoid bone (Figures 1A,B). The upper part of the medial edge is formed by the lateral surface of the optic strut, and the lower part by the body of the sphenoid bone (Figures 1A,B). The lower margin of the fissure is formed by the junction of the greater wing with the body of the sphenoid (Figures 1A,B).

The optic canal is bounded by the superior root of the lesser wing of the sphenoid bone superiorly, the optic strut inferolaterally, and the body of the sphenoid bone medially (Figures 1A,B).

The convergence portion (the proper orbital apex) is attached to a circular fibrous ring, called the common annular tendon, which serves as the tendinous origin of the rectus muscles. The common annular tendon divides the superior orbital fissure into three compartments: superolateral, central, and inferior.

### Endoscopic Endonasal Anatomy of the Orbital Apex

**Figure 2:**
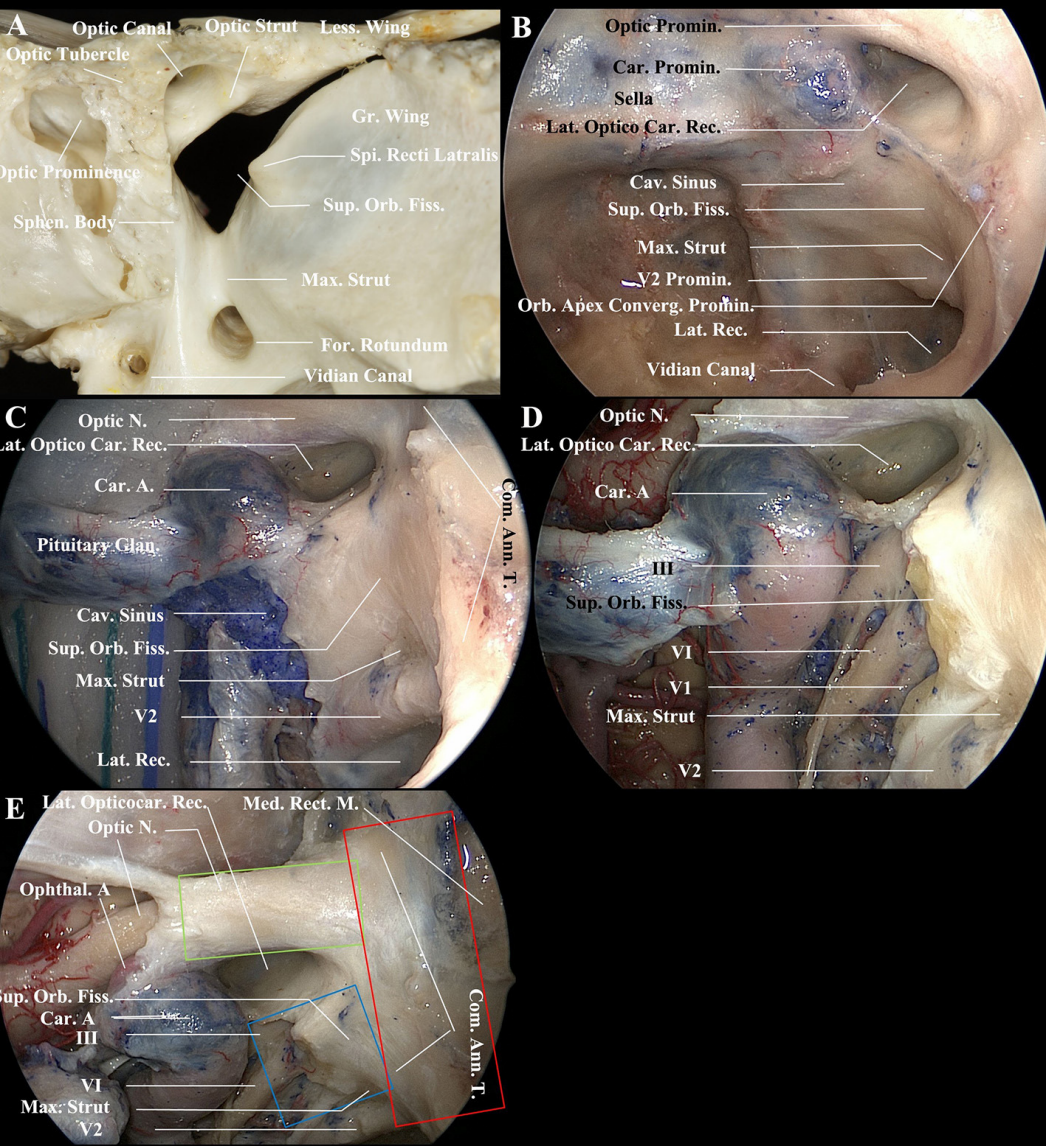
Endoscopic endonasal views of the medial wall of the orbital apex and the lateral wall of the sphenoid sinus (left side). **(A)** The osseous optic apex consists of the optic canal superomedially and the superior orbital fissure inferolaterally. The contents of the superior orbital fissure are separated from the optic nerve and ophthalmic artery, which pass through the optic canal, by the optic strut superiorly. In addition, they are separated from the maxillary division of the trigeminal nerve that courses by the foramen rotundum by the maxillary strut inferiorly. **(B)** Endoscopic endonasal view of the lateral wall of the sphenoid sinus. From superior to inferior, the lateral wall of the sphenoid sinus contains the prominences of the optic nerve, the internal carotid artery, the superior orbital fissure, and the maxillary division of the trigeminal nerve. The orbital apex convergence prominence, at which the contents of the optic canal and the superior orbital fissure converge, is located at the junction of the lateral wall of the sphenoid sinus and the orbit plate of the ethmoid bone (lamina papyracea). Between these prominences are bony recesses or depressions. The medial surface of the optic strut is the base of the lateral opticocarotid recess. The medial surface of the maxillary strut is the maxillary depression of the lateral wall of the sphenoid sinus. **(C)** View of the optic nerve, the superior orbital fissure, and the maxillary division of the trigeminal nerve after removal of the bone of the lateral wall of the sphenoid sinus. The anteroinferior compartment of the cavernous sinus, indicating the posterior boundary of the medial wall of the superior orbital fissure, was incised. The common annular tendon was indicative of the orbital apex convergence portion (the proper orbital apex). **(D)** Removal of the periosteum and periorbita of the medial wall of the superior orbital fissure and part of the cavernous sinus to identify cranial nerves III, VI, V1, and V2 and adipose tissue of the orbital apex. **(E)** A 45-degree endoscopic endonasal view of the proper orbital apex, optic nerve (intracanal segment), and superior orbital fissure contents. The optic nerve superiorly (green line box) and the superior orbital fissure contents inferiorly (blue line box) converge into the convergence portion (the proper orbital apex) anteriorly (red line box). The configurations of these three structures form a π-shaped *configuration after* clockwise rotation of 90 *degrees*. A., Artery; Ann., Annual; Car., Carotid; Cav., Cavernous; Com., Common; Converg., convergence; Fiss., Fissure; For., Foramen; Glan., Gland; Gr., Greater; Lat., Lateralis; Less., Lessor; M., Muscle; Med., Medial; Max., Maxillary; N., Nerve; Orb., Orbital; Promin., Prominence; Rec., Recess; Rect., Rectus; Sphen., Sphenoid; Spi., Spina; Sup., Superior; T., Tendon.

Understanding the anatomy of the lateral wall of the sphenoid sinus remains crucial in EEDCFA (Figure 2A). From superior to inferior, the lateral wall contains the prominences of the optic nerve, the internal carotid artery, the superior orbital fissure, and the maxillary division of the trigeminal nerve (V2) (Figures 2B,C). In addition, there is a bulge at the junction of the lateral wall of the sphenoid sinus and the orbit plate of the ethmoid bone (lamina papyracea) (Figures 2B–D). We have called this bulge the orbital apex convergence prominence because it is the convergence portion of the orbital apex, in which the contents of the optic canal and of the superior orbital fissure converge into the orbit. Beneath the convergence prominence is the common annular tendon (Figure 2C). The contour of these three prominences (the optic nerve, the superior orbital fissure, and the orbital apex convergence) forms a π configuration following a 90-degree clockwise rotation. Between these prominences are bony recesses or depressions, namely, the lateral opticocarotid recess, the depression between the cavernous sinus apex and the maxillary nerve, and the lateral sphenoid recess between the maxillary division of the trigeminal nerve and the vidian canal. The superior orbital fissure is seen as a broad prominence on the upper part of the lateral wall (Figures 2B,C). Its identification is facilitated by locating the opticocarotid recess superiorly and the bulge of the maxillary division of the trigeminal nerve inferiorly (Figures 2B,C). The posterior boundary of the superior orbital fissure is the anteroinferior compartment of the cavernous sinus (Figures 2B–D). Following the removal of the bone of the lateral wall of the sphenoid sinus, the optic nerve, the superior orbital fissure, and the maxillary division of the trigeminal nerve could be identified (Figures 2C,D). The periosteum and periorbita of the medial wall of the superior orbital fissure and part of the cavernous sinus were removed to identify cranial nerves III, VI, V1, and V2 and the adipose tissue of the orbital apex (Figure 2D). The optic nerve superiorly and the superior orbital fissure contents inferiorly converge into the proper orbital apex anteriorly. These three structures compose a π-shaped configuration (Figure 2E).

### Endoscopic Endonasal Simultaneous Decompression of the Optic Nerve, Superior Orbital Fissure, and Proper Orbital Apex

**Figure 3:**
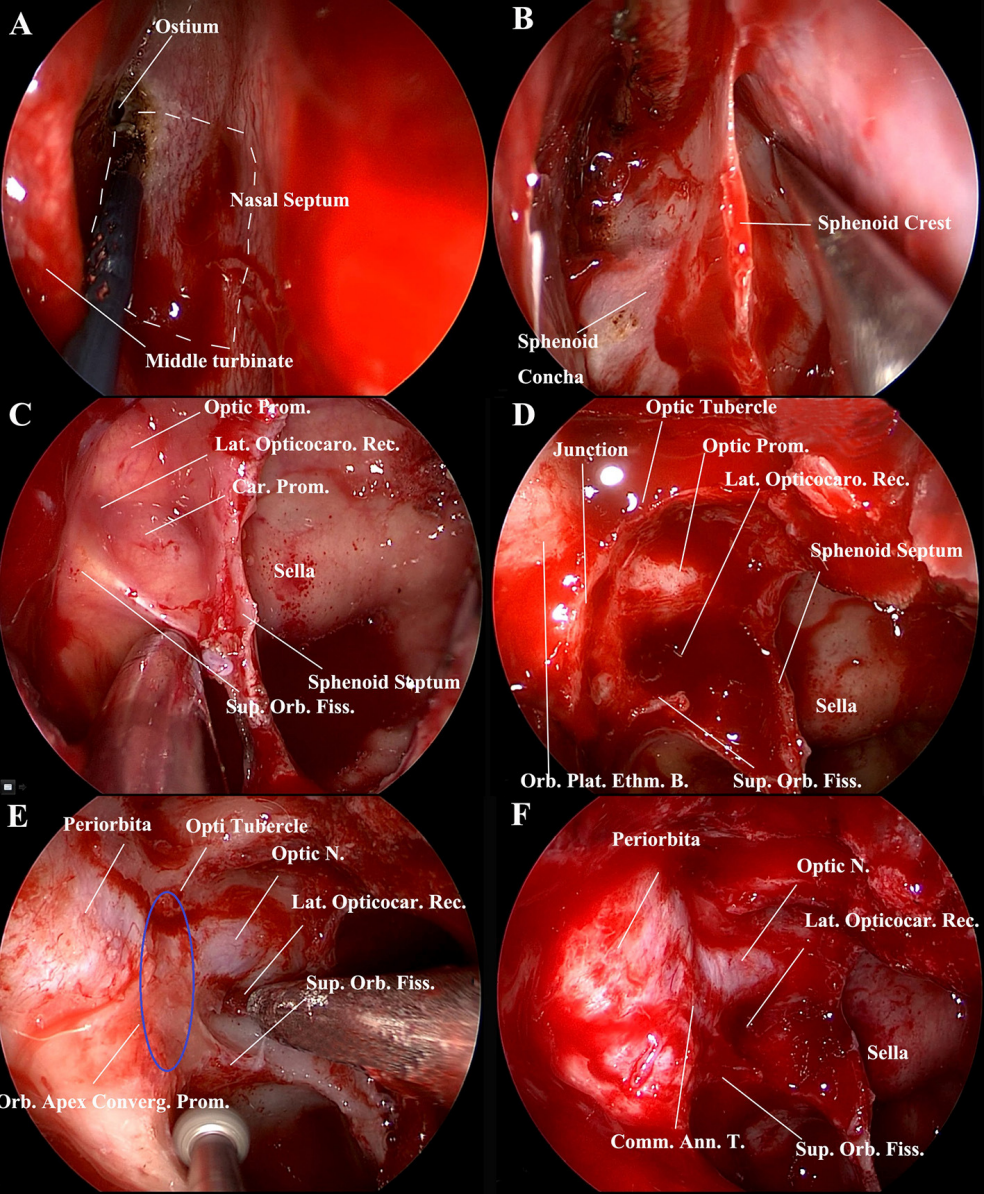
Endoscopic endonasal procedure for decompression of the orbital apex, including the medial walls of the optic canal the superior orbital fissure, and the orbital apex convergence portion (proper orbital apex) (right side). **(A)** The nasal cavity was decongested with cotton pledgets soaked in 1:10,000 adrenaline, and landmarks, namely, the middle turbinate, superior turbinate, and ostium of the sphenoid sinus, were identified. A free mini posterior nasoseptal flap (white dotted lines) was harvested for reconstruction. **(B)** Posterior nasal septostomy and anterior sphenoidal wall resection. The contralateral mucosa of the anterior face of the sphenoid sinus was incised for a binostril approach. **(C)** The wide opening of the anterior face of the sphenoid facilitates access by the endoscope and up to three instruments simultaneously. Panoramic visualization of surgical landmarks aided orientation during the surgical procedure. Landmarks of the lateral wall of the sphenoid sinus included the optic prominence, the opticocarotid recess, and the superior orbital fissure prominence. **(D)** Endoscopic endonasal view of the medial wall of the orbital apex, namely, the optic prominence, the superior orbital fissure prominence, and the orbital apex convergence prominence, with the latter being the junction of the lateral wall of the sphenoid sinus and the orbit plate of the ethmoid bone. **(E)** Drilling of the bones of the medial wall of the optic canal, superior orbital fissure, and orbital apex convergence prominence. The orbital apex convergence prominence coursed from the optic tubercle superiorly to the junction of the orbital plate of the ethmoid bone and the lateral wall of the sphenoid sinus inferiorly (blue elliptic line). **(F)** Endoscopic view of the optic nerve (intracanal segment), superior orbital fissure, and orbital apex convergence portion (proper orbital apex). The optic nerve superiorly and the superior orbital fissure inferiorly converge into the convergence portion (proper orbital apex). The contour of these three structures forms a π-shaped configuration after 90-degree rotation. The optic sheath, the periosteum of the superior orbital fissure, and the periorbita of the orbital apex were intact. Ann., Annual; B., Bone; Car., Carotid; Com., Common; Converg., Convergence; Ethm., ethmoidal; Fiss., Fissure; Lat., Lateralis; Orb., Orbital; Plat., plate; Prom., Prominence; Rec., Recess; Sphen., Sphenoid; Sup., Superior; T., Tendon.

The patient was placed under general anesthesia and orotracheal intubation. The nasal vestibules were prepped with an iodine solution. The draping left both eyes exposed in the operative field allowing intraoperative evaluation of the globes if required. Following decongestion of the nasal cavity with cotton pledgets soaked with 1:10,000 adrenaline, the landmarks of the nasal cavity on the right side were identified (Figure 3A). The ipsilateral middle turbinate and superior turbinate were out-fractured to provide good access. An ipsilateral free mini posterior nasoseptal flap was harvested for reconstruction. The contralateral mucosa of the anterior face of the sphenoid sinus was incised for a binostril approach, followed by posterior septectomy and anterior sphenoidal wall resection (Figure 3B). The ipsilateral anterior face of the sphenoid was opened widely to facilitate simultaneous access of the endoscope and up to three instruments. An ipsilateral anterior and posterior ethmoidectomy was performed to better visualize the lateral walls of the sphenoid sinus and orbital apex. This allowed recognition of the landmarks of the lateral wall of the sphenoid sinus and the orbital apex, namely, the optic prominence, opticocarotid recess, and superior orbital fissure prominence (Figures 3C,D). The maxillary branch of the trigeminal nerve (V2) and the vidian canal could be identified superolaterally and inferomedially, respectively, on the lateral recess of a well-pneumatized sphenoid sinus (Figures 3D,E). Also visible was the orbital apex convergence prominence, which coursed from the optic tubercle superiorly to the junction of the orbital plate of the ethmoid bone with the lateral wall of the sphenoid sinus inferiorly (Figures 3D,E). The bone of the medial wall of the optic canal, superior orbital fissure, and the orbital apex convergence prominence was thinned with a high-speed diamond drill under constant irrigation (Figures 3D,E). The thinned bone was gently outfractured with a curette and Kerrison rongeur. The optic nerve superiorly and the superior orbital fissure contents inferiorly were found to converge into the proper orbital apex anteriorly. These three structures formed a π-shaped configuration (Figure 3F). The optic sheath, the periosteum of the superior orbital fissure, and the periorbita of the orbital apex were left to be intact (Figure 3F). The free mini nasoseptal flap was placed over the optic nerve and superior orbital fissure. Nasopores were placed in the nasal cavity on either side. Ceftriaxone was administrated during the period of nasal packing. The vision was monitored, as was any hemorrhagic nasal discharge during the first 3 post-operative days. An orbital CT scan was performed immediately after surgery to assess the extent of decompression. Patients were usually discharged after 4–5 days.

### Clinical Outcomes of Two Patients

A total of two patients with TOAS underwent EEDCFA. The extraocular mobility functions and visual acuity of these two patients improved following decompression procedures. No surgical complications occurred. Demographic and pre- and post-operative visual and extraocular mobility functions of two cases with TOAS were listed in Table 1.

**Table 1:** Demographic and pre- and post-operative visual and extraocular mobility functions of two cases with TOAS.

	**Sex**	**Age**	**Side**	**Follow-up period**	**Visual function**		**Extraocular mobility**		**Surgical complication**
					**Pre-op**	**Post-op**	**Pre-op**	**Post-op**	
Case1	m	31	Right	12 months	No LP	LP	CO	Normal	None
Case 2	m	30	Right	8 months	No LP	LP	CO	Normal	None

*LP, Light perception; CO, complete ophthalmoplegia*.

### Illustrative Case

**Figure 4:**
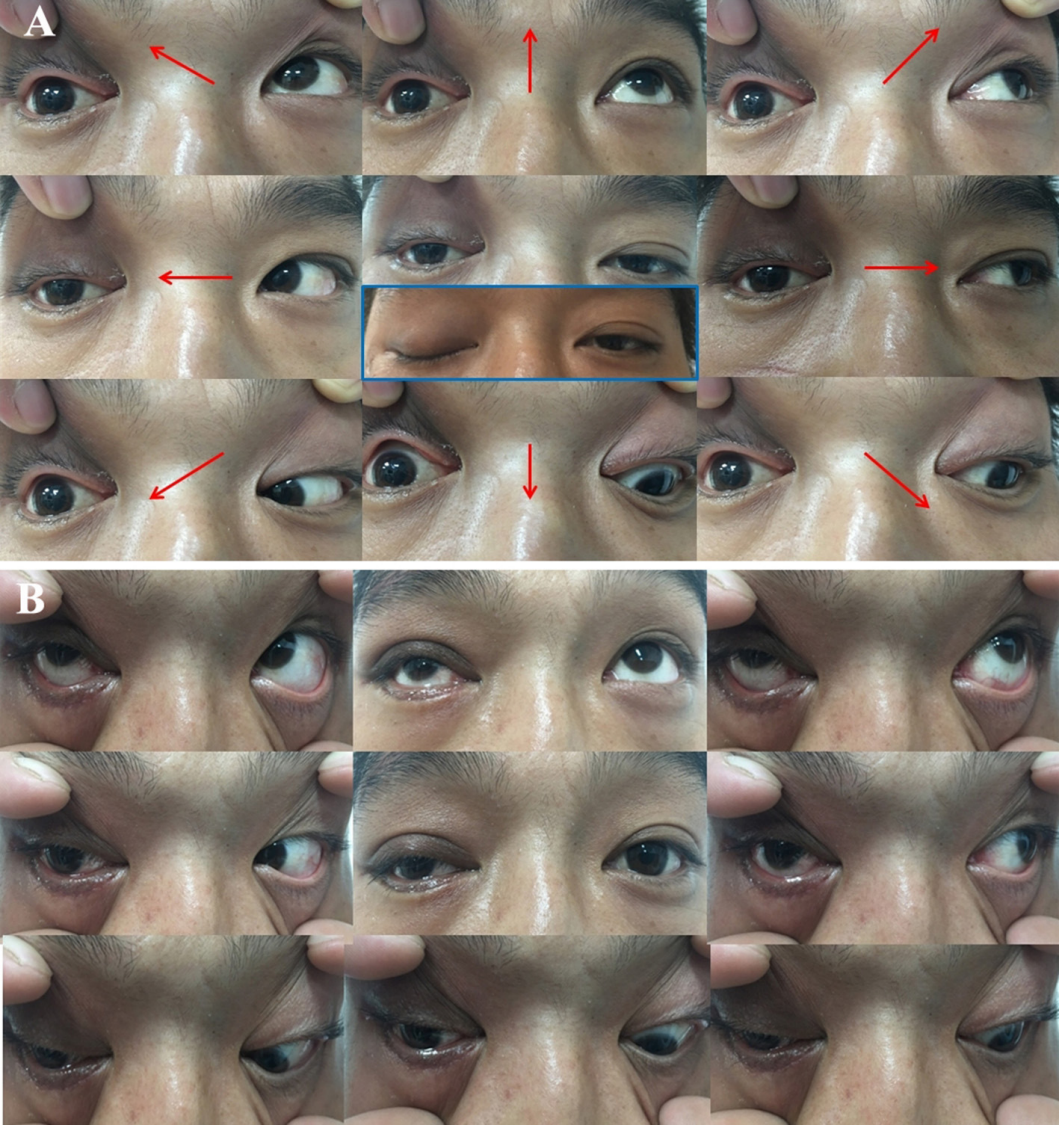
Photographs of gaze positions in nine directions before **(A)** and 3 weeks after **(B)** surgery, namely, primary gaze (middle), elevation (top middle), depression (bottom middle), dextroversion (middle left), levoversion (middle right), dextroelevation (top left), dextrodepression (bottom left), levoelevation (top right), and levodepression (bottom right) views. **(A)** Complete ophthalmoplegia and ptosis were observed on the right side before surgery. **(B)** Partial recovery of ptosis, along with infraduction and adduction, was observed 3 weeks after surgery.

**Figure 5:**
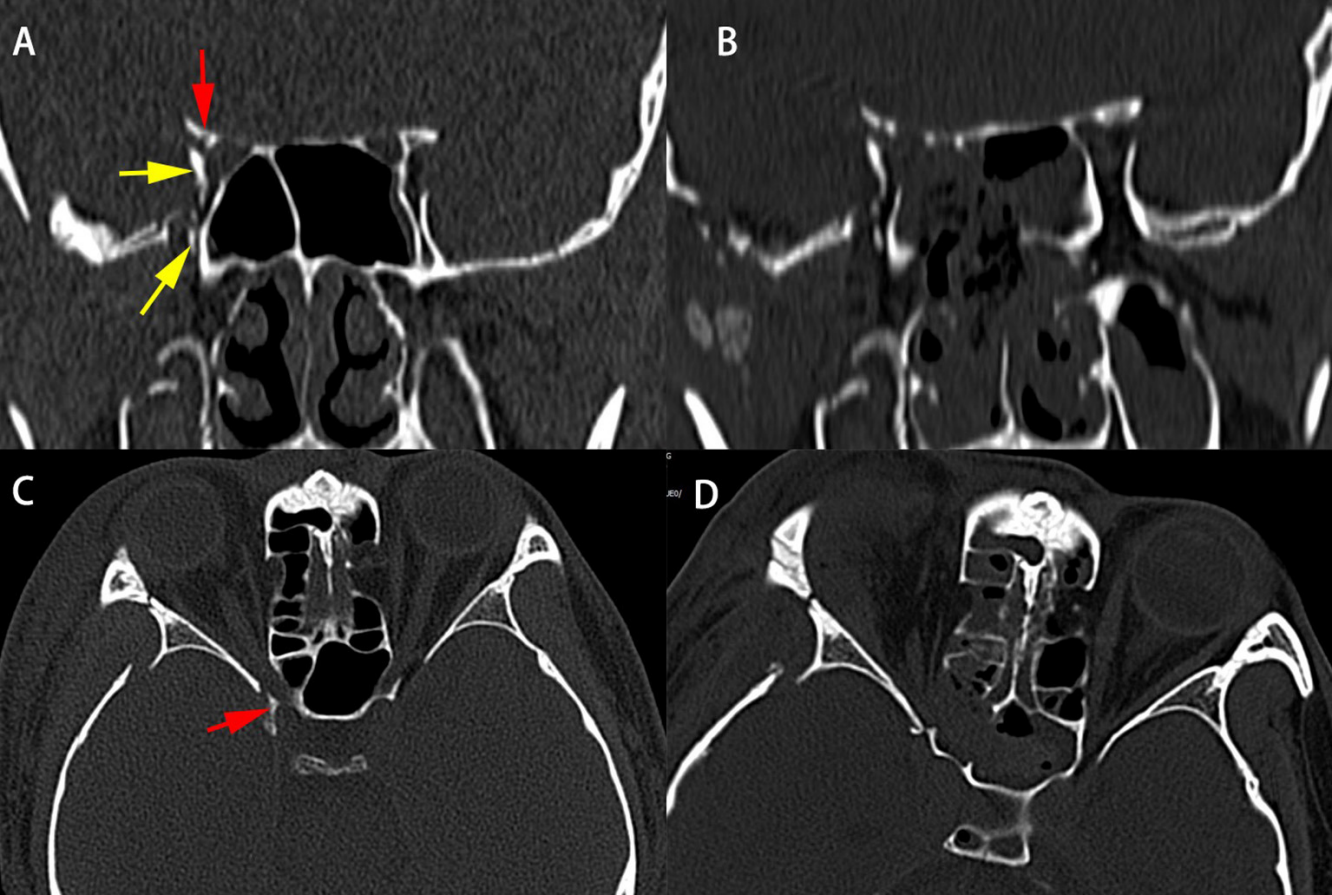
CT scans of patient 1 before **(A,C)** and immediately after **(B,D)** surgery. Pre-operative coronal **(A)** and axial **(C)** CT scans showing compression of the superior orbital fissure by the medially displaced fracture segment of the greater wing of the right sphenoid (yellow arrowheads) and the optic canal (red arrowheads). Post-operative coronal **(B)** and axial **(D)** CT scans illustrate the decompression of the optic nerve, superior orbital fissure, and proper orbital apex.

A 31-year-old male was struck by a falling steel plate at his workplace on the right side of his head. He was taken to a local clinic, where his wound was debrided and sutured. However, the bone fracture was not treated. Two weeks later, he was admitted to the ophthalmology department of our hospital. His main complaint was a complete loss of vision in his right eye and diplopia. Ophthalmologic examination revealed no perception of light, direct and indirect light reflex defects, and complete ophthalmoplegia of his right eye (Figure 4A). Administration of high-dose glucocorticosteroid was ineffective, and he was referred to our department for further treatment. Orbital CT scans showed that the orbital apex on the right side was directly compressed by displaced fracture segments of the greater wing of the sphenoid bone (Figures 5A,C), resulting in a diagnosis of TOAS. He, therefore, underwent EEDCFA. He showed a slight movement of his right eyeball on post-operative day 1, although the eyelid remained immotile. Post-operative CT scans revealed that the medial walls of the superior orbital fissure, optic canal, and posterior orbit had been successfully removed (Figures 5B,D). After 3 weeks, infraduction, superduction, and abduction were observed, whereas adduction was not (Figure 4B). Six months after surgery, he showed a full range of eye movements in all directions. Visual acuity of his right eye 10 months after surgery had improved to light perception. He experienced no surgical complications.

## Discussion

### Endoscopic Endonasal Anatomy of Orbital Apex Region

The concept of the orbital apex was controversial. It has been defined as the most posterior orbit, not including the contents of the superior orbital fissure and optic canal (10, 11, 13). Alternatively, it has been reported to consist of the contents of the superior orbital fissure and the optic canal (14, 15). We thought the orbital apex consists of three portions both anatomically and clinically: the optic canal contents superomedially, the superior orbital fissure contents inferolaterally, and the proper orbital apex or convergence portion anteriorly. The superior orbital fissure appears as a broad prominence on the upper part of the lateral wall of the sphenoid sinus. The optic nerve is a prominence located superiorly to the superior orbital fissure prominence. The orbital apex convergence bulge is a novel important landmark, coursing vertically from the optic tubercle superiorly to the junction of the orbital plate of the ethmoid bone along with the lateral wall of the sphenoid sinus inferiorly. The optic nerve prominence superiorly, the superior orbital fissure prominence inferiorly and the orbital apex converge prominence anteriorly form a π-shaped configuration indicating the orbital apex. Successful endoscopic decompression surgery requires thorough knowledge of these anatomic landmarks of the orbital apex.

### EEDCFA

#### Management of TOAS Remains Unclear

Conservative management and close observation without surgical intervention may be sufficient (16, 17). Megadoses of corticosteroids may be successful in the absence of evidence of bone dislocation (18, 19), with steroid treatment associated with a better likelihood of neurologic recovery than observation alone (20). Surgical decompression is indicated, however, in patients with evidence of a displaced osseous fracture with impingement at the orbital apex (21, 22).

The traditional approaches to the medial orbital apex include medial orbitotomy with or without lateral orbitotomy (23), a transantral Caldwell–Luc approach (24), and pterional craniotomy from either the ipsilateral or contralateral side (25). The endoscopic endonasal approach to the orbit apex and wall is increasingly used (26–29). Zhou et al. (1) reported endoscopic endonasal optic canal and proper orbital apex decompression for 31 patients with TOAS. Nineteen of 31 patients gained improvement of best-corrected visual acuity after surgery, seven of 31 gained 20/20, and visual field showed improvement in 20 patients. Ptosis and ophthalmoplegia of all patients recovered in various degrees; diplopia was also relieved relatively. In this article, however, authors just performed optic canal and proper orbital apex decompression, they did not perform superior orbital fissure decompression. Jin et al. (2) reported 13 cases of TOAS underwent decompression of superior orbital fissure and optic canal *via* an open microsurgical approach. The visual and extraocular mobility function of the patient improved to various extents. Imaizumi et al. (4) reported a patient with TOAS whose visual and extraocular function improved quickly after emergency decompression surgery of the superior orbital fissure and the optic canal by the epidural approach using a frontotemporal craniotomy.

To our knowledge, this study is the first report on simultaneous decompression of the optic canal, superior orbital fissure, and proper orbital apex for TOAS *via* endoscopic endonasal approach. The two patients had satisfactory recovery of extraocular motility and improvement of visual acuity. No surgical complication was reported.

#### Limitations

EEDCFA was undertaken in two cases in our institute. We will adopt this technique in a greater volume of patients with TOAS.

## Conclusion

The π-shaped configuration formed by the optic nerve prominence, the superior orbital fissure prominence, and the orbital apex converge prominence indicates the orbital apex and it is an important landmark for EEDCFA. EEDCFA is feasible, effective, and safe for patients with TOAS caused by direct compression of displaced fracture segments.

## Data Availability Statement

The raw data supporting the conclusions of this article will be made available by the authors, without undue reservation.

## Ethics Statement

The studies involving human participants were reviewed and approved by Ethics Committee of China-Japan Friendship Hospital. The patients/participants provided their written informed consent to participate in this study.

## Author Contributions

JL conceived and designed the surgical technique. JZ and YW performed enrollment of cases and collected the clinical data. JL and JZ analyzed the data and wrote the paper. ZW, RL, ZC, YZ, JH, and DY revised the paper critically for important intellectual content. All authors read and approved the final manuscript.

## Funding

JL was supported by Natural Science Foundation of Beijing (7212090).

## Conflict of Interest

The authors declare that the research was conducted in the absence of any commercial or financial relationships that could be construed as a potential conflict of interest.

## Publisher's Note

All claims expressed in this article are solely those of the authors and do not necessarily represent those of their affiliated organizations, or those of the publisher, the editors and the reviewers. Any product that may be evaluated in this article, or claim that may be made by its manufacturer, is not guaranteed or endorsed by the publisher.
